# Structural and Mechanical Analysis of Individual Mineralized Collagen Fibrils Using In Situ Transmission Electron Microscopy

**DOI:** 10.1021/acsnano.6c00964

**Published:** 2026-03-19

**Authors:** Tatiana Kochetkova, Stephanie M. Ribet, Lilian M. Vogl, Daniele Casari, Rohan Dhall, Philippe K. Zysset, Andrew M. Minor, Peter Schweizer

**Affiliations:** † ARTORG Center for Biomedical Engineering Research, 27210 University of Bern, Bern, CH 3010, Switzerland; ‡ National Center for Electron Microscopy (NCEM), The Molecular Foundry, 682791 Lawrence Berkeley National Laboratory, Berkeley, California 94720, United States; § Department of Materials Science and Engineering, University of California Berkeley, Berkeley, California 94720, United States; ∥ Max Planck Institute for Sustainable Materials, Düsseldorf 40237, Germany; ⊥ Laboratory for Mechanics of Materials & Nanostructures, 28501 Empa - Swiss Federal Laboratories for Materials Science and Technology, Thun, CH 3603, Switzerland

**Keywords:** mineralized collagen fibril, MTLT, TEM, EDX, 4D-STEM, tensile test

## Abstract

Bone serves as an example of nature’s architectured material with its characteristic blend of strength and toughness, all at a lightweight design. Given the hierarchical nature of these materials, it is essential to understand the governing mechanisms and organization of their constituents across length scales for bioinspired structural design. Despite recent advances in transmission electron microscopy (TEM) that have allowed us to witness the hierarchical arrangement of bone at micro-down to the nanoscale, we are still missing the details about the structural organization and mechanical properties of the main building blocks of bone—mineralized collagen fibrils (MCFs). Here, we present a method to extract individual MCFs from nature’s model material, mineralized turkey leg tendon, using a dropcasting procedure. By isolating the MCFs onto TEM supporting grids, we visualized the arrangement of organic and mineral phases within individual MCFs at the nanoscale. Using a four-dimensional scanning transmission electron microscopy (4D-STEM) approach, the orientation of individual mineral crystals within the MCFs was examined. Furthermore, we conducted in situ tensile experiments, revealing exceptional tensile strains of at least 8%, demonstrating the intricate relationship between structural organization and the mechanical behavior of MCFs. These insights into the ultrastructure of mineralized tissue building blocks, as well as the proposed sample-extraction method compatible with in situ mechanical testing, provide a strong basis for research into nature-inspired material design.

## Graphical abstract

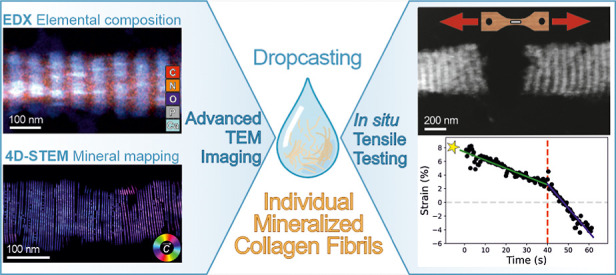

## Introduction

Bone is a complex natural material exhibiting an exceptional combination of strength and toughness while being lightweight. These remarkable material properties are triggered by the evolutionary adaptation of bone to provide mechanical support and organ protection in the vertebrates.^1^ While bone is composed of ductile organic and brittle inorganic phases, the hierarchical arrangement of the constituents from macroscale down to the nanoscale gives rise to the outstanding mechanical properties (Figure 1).

**Figure 1:**
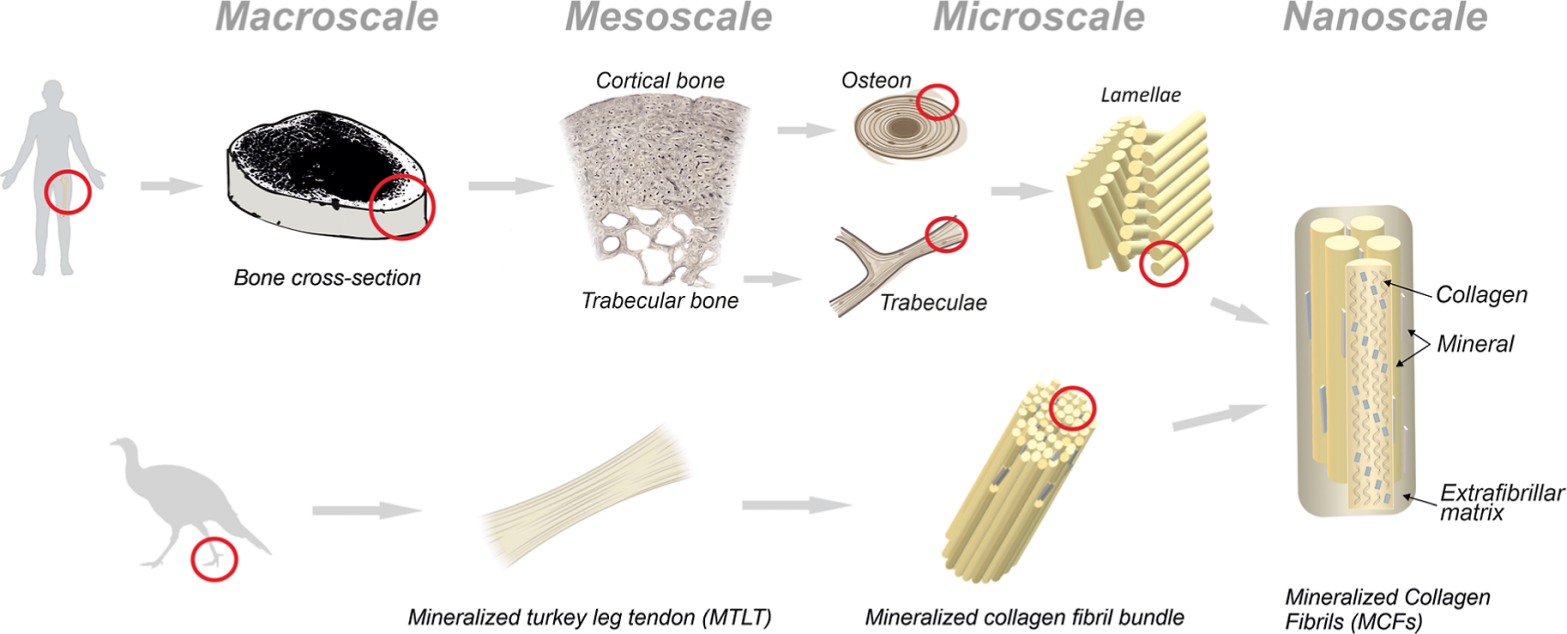
Human lamellar bone exhibits complex hierarchical organization spanning from macro- to nanoscale levels. Mineralized collagen fibrils (MCFs) are considered the building blocks of bone, made of staggered collagen tropomolecules with intra- and extramolecular inclusions of the mineral agglomerates. On the other hand, the mineralized tendon from the turkey leg is made of a uniaxial bundle of MCFs, making it an attractive model to study the building blocks of bone—the MCFs.

Mineralized collagen fibrils (MCFs) are the building blocks of bone tissue, reaching about 30–300 nm in diameter and up to hundreds of micrometers in length. MCFs are formed from staggered arrays of tropocollagen molecules intertwined with hydroxyapatite nanocrystals, stabilized by hydrogen bonds and cross-links.^2,3^ Crystal plates of hydroxyapatite (HA) assemble in the gap between the molecules, reaching tens of nanometers in length and 1–2 nm in thickness. This staggered arrangement creates an observable periodicity within the MCF known as the D-band with the reported repetition step of 67 nm.^4,5^

Direct observation of mineralized collagen fibrils (MCFs) in lamellar bone requires nanometer-scale resolution, strong collagen–mineral contrast, and, ideally, preservation of the native hydrated structure. Recent studies have employed high-resolution TEM and STEM for mineral–collagen imaging,^6,7^ electron tomography for nanoscale 3D reconstructions,^8,9^ FIB–SEM for volumetric mapping of mineral organization,^10,11^ and synchrotron-based conventional SAXS/WAXS or even their tensor tomography modalities for orientation and density mapping.^12–14^ Across these modalities, common limitations stem from sample preparation (chemical dehydration, embedding, staining, and etching), which can alter native fibril morphology; trade-offs between resolution and field of view; and restricted imaging volumes in high-resolution 3D methods. As a result, while the hierarchical organization of MCFs within bone is well documented, direct visualization of fully isolated, intact MCFs in their native state remains scarce, leaving key aspects of the individual fibril geometry and mineralization unresolved.

Other studies have attempted to synthesize MCF-like structures by mineralizing nonmineralized collagen fibrils in vitro using biomimetic approaches such as polymer-induced liquid-precursor mineralization, simulated body fluid immersion, or controlled calcium phosphate precipitation.^15–17^ While these synthetic constructs often reproduce the expected nanoscale organization of apatite platelets within the collagen gap zones, they inevitably lack the natural developmental and compositional context of bone. Consequently, their structure and properties, although informative, cannot fully substitute for direct observations of native MCFs.

A new methodological approach is therefore required—one that enables in situ, high-resolution visualization of natural MCFs in their unaltered state, preserving both mineral and collagen phases without destructive sample preparation. Such an approach would close the critical knowledge gap between synthetic models and the true nanoscale architecture of bone.

Here, we propose the mineralized turkey leg tendon (MTLT) as a source of MCFs, which is more straightforward to extract and easily accessible. MTLT is composed of densely packed collagen fibrils, which are strongly aligned with the tendon axis, in contrast to the complex structure of mammalian cortical bone.^18,19^ The chemical composition as well as the crystal organization in MTLT closely approximate those found in bone,^20–25^ with just the differences in the mineralization process and more crystalline appearance of the intrafibrillar mineral being reported.^26^ Following the structural and compositional similarities, MTLT has also been used as a valuable model to study the mechanical properties of bone at the microscale.^19,27–29^ However, whether this can be extended to nanoscale studies of individual MCFs remains to be investigated.

This work pursues two goals: (i) TEM analysis of isolated MCFs and (ii) in situ TEM observations of the MCFs’ deformation behavior. For this, we develop a novel protocol for the MCFs extraction and image them using the high-resolution TEM techniques. Ultimately, the isolated MCF imaging allows the in situ mechanical tests, elucidating the fundamental processes of fibril deformation, and has the potential to pave the way for creating innovative bioinspired nanostructured materials.

## Results

### Imaging the Individual MCF

The drop-casting protocol enables the extraction of MCFs from the bulk MTLT sample, preserving their native arrangement. For this, individual MCFs were extracted from MTLT samples using mechanical splitting along the main axis with a surgical scalpel, followed by ultrasonification of the MTLT fragments. The supernatant with the intact MCFs was then transferred to the TEM grids through dropcasting (Figure 2). Two types of grids were used: (i) a copper TEM grid with lacey carbon films for static imaging and (ii) copper tensile stripes with a support film in the central window for tensile tests.

**Figure 2:**
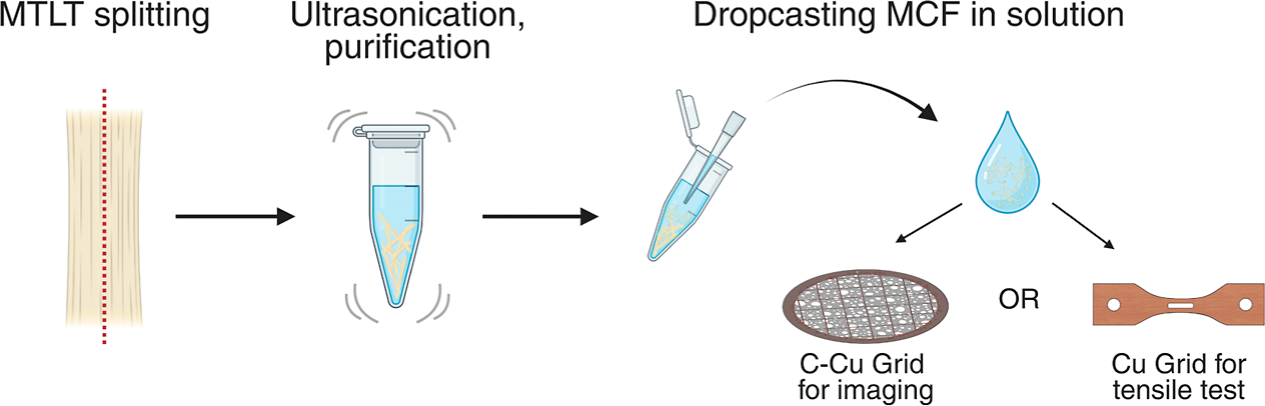
Hydrated mineralized turkey leg tendons (MTLT) were split along the main axis and ultrasonicated; the resulting solution with mineralized collagen fibrils (MCFs) is dropcasted on (i) a copper TEM grid with lacey carbon films for imaging or (ii) a copper tensile stripe with support film in the central window. Figure created using BioRender.com.

### Origins of Nanoscale Periodicity

Individual MCFs were observed on the lacy carbon support film of the TEM grids, with their characteristic compositional contrast clearly resolved in both high-angle annular dark-field (HAADF) STEM images and corresponding energy-dispersive X-ray spectroscopy (EDX) elemental maps (Figure 3). The periodic arrangement of mineral phases was evident in both imaging modes, yielding an average D-period of 68.6 ± 0.2 nm, as determined from the elemental intensity profiles of five MCFs (Figure S1). This value is slightly larger than the canonical 67 nm reported for native collagen.^4^ MCFs exhibited varying degrees of mineralization, with calcium-to-phosphorus (Ca/P) ratios ranging from 0.26 to 1.63; the upper value being close to the theoretical Ca/P ratio of 1.67 for stoichiometric hydroxyapatite.^30^ Analysis of a limited data set (*n* = 5) indicated a negative correlation between the D-period and Ca/P ratio (*R* ^2^ = 0.78, Figure S2), suggesting that higher mineral content is associated with a slight reduction in the fibril’s D-period.

**Figure 3:**
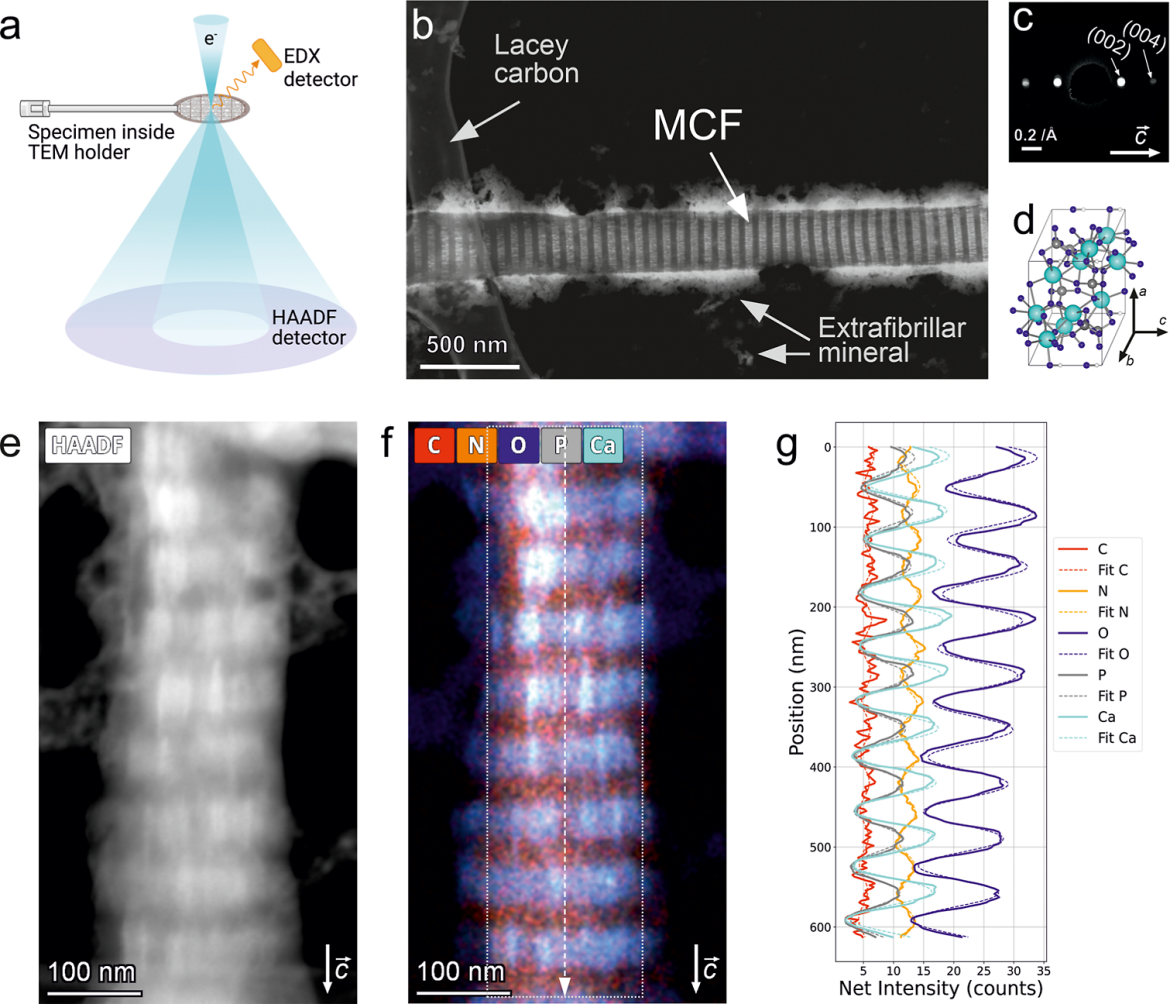
MCF appearance in TEM. (a) Schematic of TEM imaging with an energy-dispersive X-ray spectroscopy (EDX) detector and a high-angle annular dark-field (HAADF) detector. The overview HAADF STEM image (b) demonstrates visible periodicity, while the electron diffraction (c) highlights the preferred crystallographic orientation of the HA (d) along the main fiber axis. Close-up HAADF (e) captured the periodicity of the MCF organization along the main axis, followed by periodic compositional distribution as visible from EDX maps (f) and their net intensity fits (g).

### Crystal Localization and Orientation

Four-dimensional scanning transmission electron microscopy (4D-STEM) was used to probe the nanoscale structure of the mineralized collagen fibrils. In this method, a converged electron nanobeam is raster-scanned across the specimen, and a pixelated detector records a diffraction pattern at each probe position, enabling the reconstruction of spatially resolved structural maps even from beam-sensitive materials like MCFs.^31^ The 4D-STEM data sets obtained from individual fibrils revealed distinct diffraction features corresponding to crystalline mineral domains embedded within the collagen matrix.

To assess mineral orientation, we applied flow line mapping relative to the crystallite *c*-axis (the (002) reflection). The detailed data processing and reconstruction pipeline is summarized in Figure S3. In this approach, the angular position of the (002) diffraction spot is measured at each scan pixel, and the resulting orientation vectors are plotted as continuous lines across the real-space map, tracing the in-plane orientation of the crystallites (Figure 4). In six out of eight 4D-STEM scans, the flow line maps demonstrated moderate alignment of the hydroxyapatite *c*-axis to the MCF backbone with an angular deviation of 2 ± 12° (Scans 1–6, Figure S4). The remaining two scans revealed a broad range of hydroxyapatite orientations, suggesting a strong influence of the extrafibrillar mineral (Scans 7 and 8, Figure S4). Moreover, variations in diffraction peak intensity across the scans indicated heterogeneity in local mineral content, in agreement with the variations in the Ca/P ratio measured by EDX.

**Figure 4:**
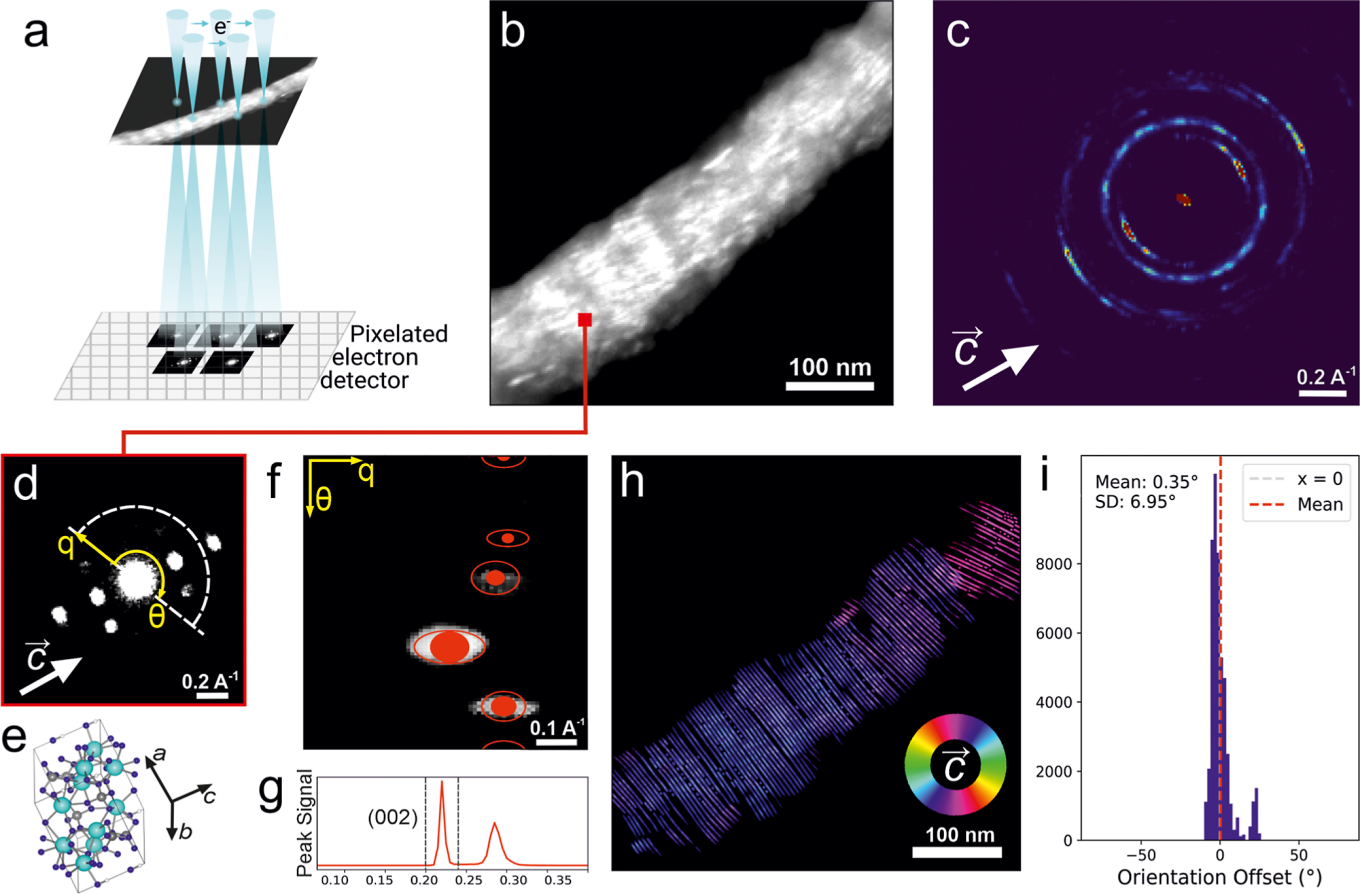
4D-STEM scan of a representative MCF. (a) Schematic of 4D-STEM imaging principles. (b) Reconstructed virtual dark-field image of an MCF fibril. (c) Mean electron diffraction from the MCF fibril with a marked *c*-axis of hydroxyapatite. (d) Local electron diffraction from a single pixel marked in the dark field image (b) and a corresponding schematic of HA crystallite orientation (e). (f) Following the polar transformation as marked in (d), diffraction peaks are fitted, and the (002) reflection is selected for the image reconstruction (g). (h) Resulting flow line map depicts the in-plane orientation of the *c*-axis of the hydroxyapatite crystallite. (i) Histogram of the orientation offset of the hydroxyapatite crystallite relative to the fibril orientation.

### Structurally Guided Mechanical Properties

In situ tensile experiments on mineralized collagen fibrils (MCFs) were performed in a double-aberration-corrected FEI Titan microscope (TEAM I) using a single-tilt straining holder equipped with a custom-designed copper tensile stripe. Dropcasted MCFs were distributed across a nitrocellulose support film in the central window of the tensile stripe. Quasistatic tensile loading was manually applied using the straining sample holder, inducing guided stretching of the support film and attached MCFs.

A total of three MCFs were imaged under tensile loading (Figures 5 and S5). All three MCFs exhibited ductile deformation under loading, with distorted crack propagation (Figures 5 and S5b) and visible deterioration of the D-period (Figure S5a). Quantitative analysis of the deformation behavior was performed on the fibril shown in Figure 5, where a clear D-period was visible throughout the entire sequence of recorded HAADF images, allowing us to track the deformation progression. This fibril exhibited crack initiation and propagation, revealing key deformation mechanisms under tensile stress. Crack nucleation occurred at the interface between the mineral-rich (gap zone, appears bright) and collagen-rich (overlap zone, appears dark) zones. Although nucleation occurred outside the initial field of view, subsequent crack growth was recorded. The crack propagated along the overlap region (dark, collagen-rich), following a distorted path within this narrow collagen-rich zone.

**Figure 5:**
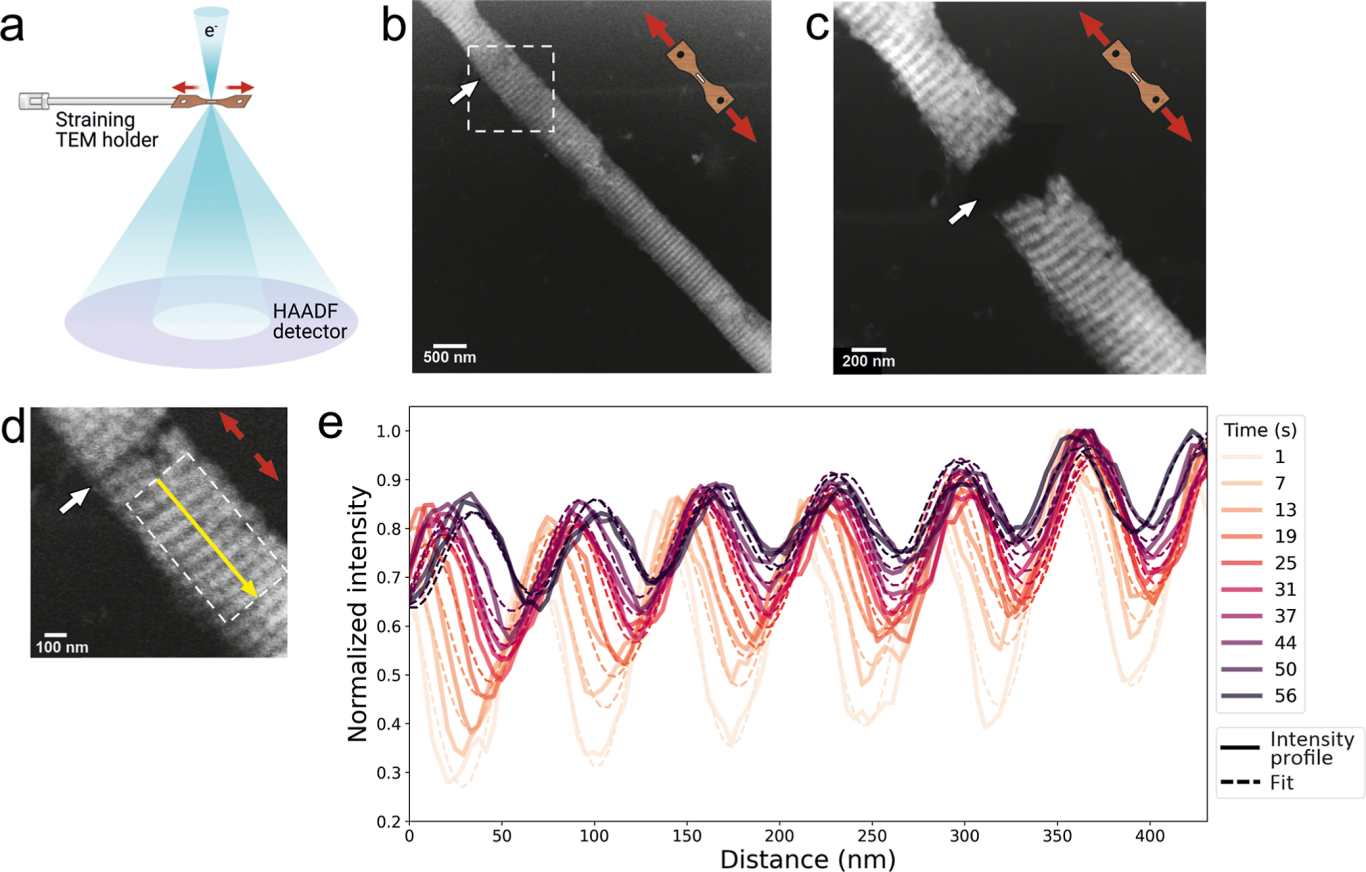
In situ tensile testing of individual MCF. (a) Schematic of tensile testing in TEM with the high-angle annular dark-field (HAADF) detector. (b) HAADF image of the MCF fibril prior to the tensile test, with the highlighted region (d) of the crack initiation. (c) HAADF image of the fibril after the separation following the tensile test. (d) Zoomed-in image of the deformation region with the marked area of intensity measurements. (e) Intensity profiles and their fits across the MCF region highlighted in (d) at selected time frames.

D-period tracking during crack progression provided further insight into the internal structural response of the fibril (Figure 6). Prior to the applied deformation (zero-strain), the D-period of the fibril near the crack opening was 69.5 ± 0.6 nm, as estimated from a series of 30 static dark-field images. Thus, the fibril was in a prestrained state before we induced the stretching. Strain relaxation was observed as the crack opened, and this relaxation continued even after complete fibril separation at 40 s, ultimately reaching the D-period of 66.6 nm. While initial crack nucleation occurred outside the imaging area, the collected data during crack progression indicated that the MCF reached a remarkable strain of 8.2%, which may have been even higher at the moment of crack nucleation. Moreover, postfailure imaging of the fracture surface revealed an irregular morphology (Figure 5c), suggesting active crack deflection at the organo–mineral interface.

**Figure 6:**
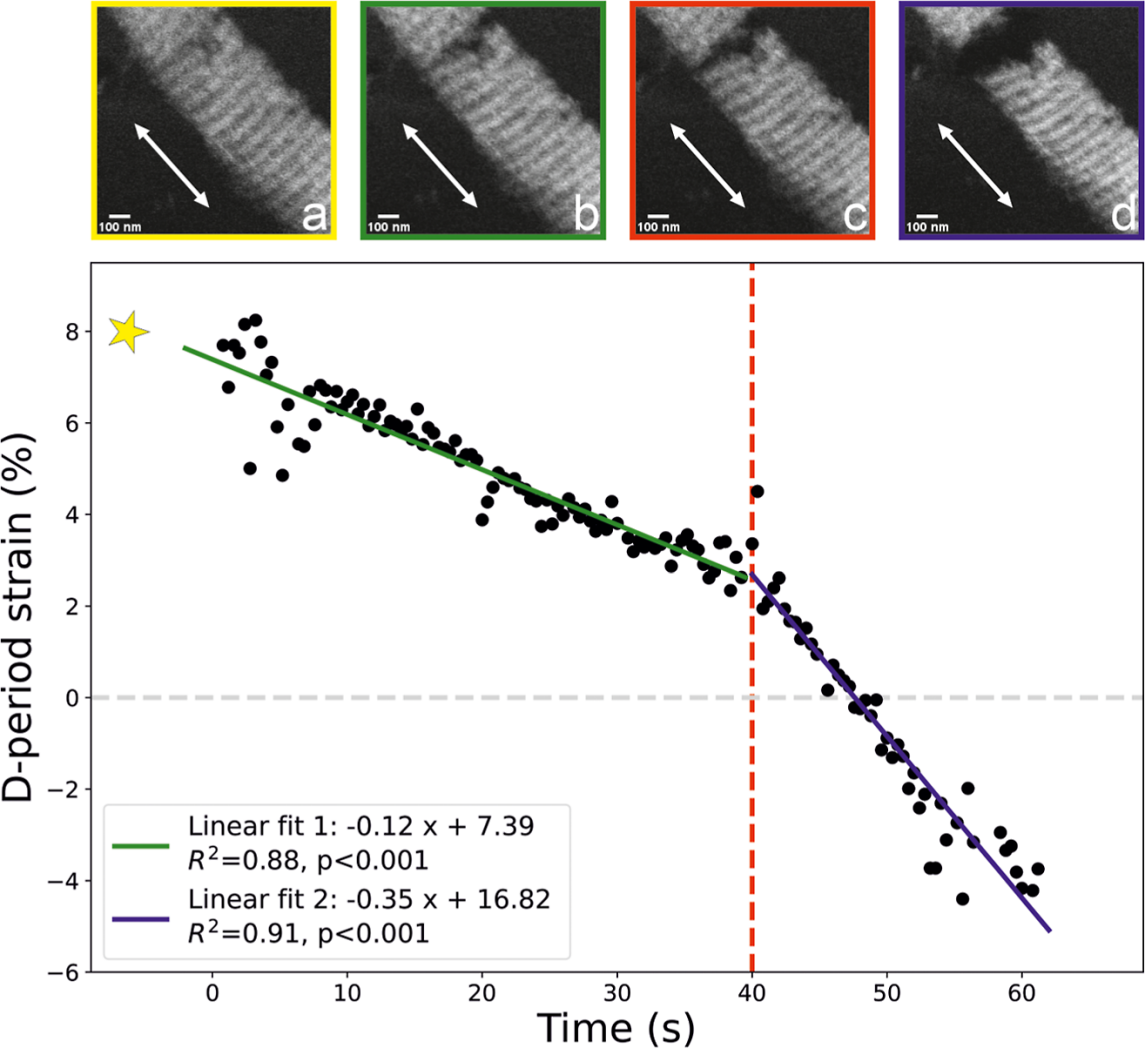
Strain relaxation during crack propagation, along with the dark field TEM image sequences of the crack appearance (a–d). The D-period of the MCF prior to applied deformation was ≈69.5 nm and was taken as a zero-strain reference for the MCF D-period strain calculation. The star indicates the estimated moment of crack nucleation, which occurred prior to imaging. The initial crack appearance is visible in (a). The first phase of the strain relaxation aligns with the crack opening shown in (b) and continues up to the final MCF rupture at about 40 s (c). The second phase of the strain relaxation continues after the final MCF separation (d), reaching negative strain. The arrow directions in (a–d) indicate the stretching direction.

## Discussion

In this work, a novel protocol for MCF extraction was developed, allowing advanced TEM analysis of individual MCFs and subsequent in situ observations of their deformation mechanics. This was only possible following the newly proposed dropcasting protocol.

Dropcasting is an established and widely utilized technique for TEM sample preparation, wherein a small volume of sample suspension is deposited onto a TEM grid and allowed to dry. This method has successfully been employed to visualize the assembly of purified collagen molecules^16,32,33^ and the formation of synthetic hydroxyapatite,^34,35^ enabling high-resolution analysis of their morphology and organization.

To the best of our knowledge, we are the first to apply drop-casting to native mineralized collagen fibrils (MCFs). While we initially attempted this procedure using bone samples, we encountered challenges as the MCFs tended to fragment into short pieces during the splitting process. However, when applying this method to mineralized turkey leg tendons, we successfully extracted longer samples exceeding 10 μm, while moderately removing noncollagenous proteins from the bulk material. In fact, this protocol can be extended to less mineralized collagen fibrils, such as those found in antler bone, as well as nonmineralized collagen fibrils extracted from soft tissues, including tendons and muscles, following a thorough purification process.

From our observations, the average D-period of mineralized collagen fibrils was 68.64 ± 0.16 nm, slightly above the canonical ∼67 nm value reported by Hodge and Petruska (1963).^5^ Although 67 nm is widely cited as a defining structural feature of type I collagen, several studies have shown that *D*-spacing varies among fibril bundles.^36,37^ This variability has been linked to native structural heterogeneity and to pathological changes, including estrogen-depletion-related osteoporosis.^38^ In our samples, the reduction in the D-period with higher Ca/P ratios may reflect both this inherent variability and a compaction effect from mineral infiltration. As mineral platelets occupy intrafibrillar gap zones and extend into overlap regions, they can restrict axial packing of collagen molecules, thus inducing axial contraction of collagen fibrils.^39^ The prestraining of the MCFs due to the vacuum exposure and possibly due to the freeze-thaw cycle might have further introduced detected alterations from the canonical 67 nm. Although no changes in the mechanical properties of MCF have been reported after repeated freeze-thaw cycles,^40^ there are no studies examining possible changes in D-period due to freezing. In this study, all samples were freeze-thawed prior to measurement, and assessing the potential impact of sample storage is of interest for future research.

Moreover, as was recently highlighted in the work of Shah,^41^ the estimated mineralization from the Ca/P ratio may only serve as a proxy for bone mineral maturity. Mainly due to the oversimplified chemistry within bone since both Ca^2+^ and PO_4_^3–^ ions are, in fact, partially substituted with Mg^2+^, Na^+^ and CO_3_^2–^. The suggested alternative would be the ratio Ca + Mg + Na/P + C, which could not be estimated from our measurements due to the low concentrations of substitutional elements beyond the EDX detection limit. Another limiting factor for the measured Ca/P ratios is the lack of standard hydroxyapatite reference measurements. Additionally, the carbon signal in the gap zones may be influenced by edge effects, further complicating the analysis.

It is important to note that mineralized collagen fibrils (MCFs), like any biological tissue, are inherently heterogeneous; thus, the native variations in mineralization are expected and in line with our observations. In the present work, samples were freshly prepared for each transmission electron microscopy modality to ensure that imaging was executed under consistent conditions immediately following extraction from the bulk material. Consequently, EDX mapping, 4D-STEM scans, and in situ stretching were conducted on different MCFs sourced from the same piece of bulk MTLT.

In this work, we used flow line mapping of 4D-STEM data to study the orientation of hydroxyapatite crystallites in MCFs.^42^ Unlike automated crystal orientation mapping (ACOM),^43–45^ where individual experimental diffraction patterns are indexed based on a known structure file, flow line analysis focused on the chosen reflection plane of the crystal. By focusing on the (002) reflection of the apatite, the alignment of the *c*-axis relative to the fibril was evaluated. With this approach, it is possible to assess the orientation of even less crystalline minerals, including amorphous calcium phosphate, that can be found within the gap regions of the bone MCF.^8,26^ Due to varying fibril thickness and heterogeneous mineralization, still not all local orientations could be resolved. Although most imaged MCFs showed minerals well-aligned with the fibrillar axis, some fibrils exhibited substantial variations in mineral orientation, indicating a strong influence from extrafibrillar minerals (Figure S4). In bulk bone tissue, extrafibrillar mineral is generally coaligned with intrafibrillar mineral and the fibril axis.^6^ Yet in our imaging of isolated MCFs, extrafibrillar mineral is likely redeposited on the fibril surface during preparation and may not reflect the native in-bulk arrangement. While intra- and extrafibrillar mineral cannot be distinguished in our 2D projection data sets, elucidating their 3D spatial organization to separate these contributions could be achieved through electron or atom probe tomography, as has been successfully applied in lamellar bone imaging.^6,8,46–48^ Building on the successful imaging of other beam-sensitive materials,^49^ 4D-STEM tomography would be of great interest for elucidating the mineral organization within individual MCFs; however, this requires transferring the measurements to cryogenic conditions or powerful postprocessing algorithms that are robust to high-noise and large defocus data.^31,50,51^ The collected 4D-STEM scans with the flow line mapping nonetheless capture the overall orientation trends within individual fibrils, and these results and analysis pipeline will be valuable for future studies aimed at understanding mineralization progression within and along single MCFs, particularly when combined with emerging liquid-TEM capabilities.^52,53^

Last but not least, we report observations from a single mineralized collagen fibril (MCF) subjected to in situ tensile testing. Notably, this represents the first direct visualization of crack propagation in an isolated MCF. The crack initiated and propagated within the collagen overlap region, characterized by lower mineral content, in agreement with numerical models predicting stress concentration in this region under uniaxial loading.^54^ The progression of the crack revealed an interplay between the organic and mineral phases of the fibril with a deflected crack path. This tropocollagen–hydroxyapatite interplay aids the energy dissipation and fracture resistance, similar to the well-documented bone toughening mechanisms at the macro-, meso-, and microscales.^55–58^ By focusing on the deformation of individual fibrils in TEM, we can begin to resolve the mechanical behavior within and between the organic and inorganic phases, providing new insights into the fundamental mechanisms of bone toughness.

D-period tracking enabled estimation of local strain along the fibril near the crack. The observed linear strain relaxation was concurrent with the crack opening and ultimately resulted in negative strain values. These negative values are a direct consequence of our definition of zero D-period strain. Here, we used the D-period of the MCF, as measured prior to applied deformation. However, the MCF appeared to be in a prestrained state. Consequently, as the fibril relaxed toward its resting state, its D-period shortened below this initial reference, registering as a negative strain. Although strains recorded during crack progression reached at least 8.2%, the total strain alteration was even greater, amounting to 12.6% when considering the entire process down to the final fibril detachment. However, the true delta strain was likely even higher since our measurements did not capture the precise moment of crack initiation when the fibril strain would have been at its peak. Our observations thus exceed existing molecular dynamics simulations, which suggest that mineralized fibrils may achieve an ultimate tensile strain of approximately 6.7%.^59^

In situ TEM tests enabled us to achieve unprecedented resolution in tracking the D-period strains. However, such measurements are inherently limited by the high vacuum exposure, meaning that prior to the tests specimens had to be dried, which affects both the mechanical properties of the organic phase and the surface chemistry of the mineral platelets. As was demonstrated in the lamellar bone-level microtensile testing, tissue hydration leads up to a 3-fold strength decrease but activates several toughening mechanisms, enabling inelastic deformation.^60^ Similarly, the hydrated micropillar tests on the lamellar bone demonstrated decreasing yield stress^61^ in comparison to the dry tests in a vacuum.^62,63^ The microscale toughness in tension is, however, expected to decrease with the specimen drying.^60^ But from our observations, the MCF crack propagation clearly exhibits toughening mechanisms, backed by the elevated strain values. Whether the hydrated MCF would exhibit even higher toughness remains to be investigated.

Unlike nonmineralized fibrils, MCFs are challenging to extract in an intact state due to their native tight packing within the mineralized matrix. In an inspirational study by Hang and Barber on an antler bone, individual fibrils protruding from a fractured surface were mechanically pulled using the AFM cantilever, providing the stress–strain curves, but without direct visualization of the underlying deformation mechanisms.^64^ At a higher structural level, MCF bundles from model tissue (MTLT) have been tested in compression in combination with in situ synchrotron imaging, revealing load-sharing mechanisms among the constituent fibers.^27^ Beyond these examples, no prior work has demonstrated in situ mechanical testing of individual MCFs, largely because such experiments are considered to be technically impractical.

In the microscale bone tissue mechanics, recent advances have enabled testing of micron-sized specimens in uniaxial tension,^60,65^ compression,^23,61–63,66–69^ and other complex deformation fields.^70^ However, the intricate sample preparation required for these approaches makes their application to isolated MCFs highly improbable. In contrast, numerous studies have investigated the mechanics of nonmineralized collagen fibrils using atomic force microscopy (AFM) or microelectromechanical systems (MEMS) under various loading modes (nanoindentation, bending, tension)^71^ and environmental conditions (air-dried, humidified, or submerged in aqueous solutions of varying ionic strength and pH).^71^ Potentially, these testing approaches could be extended to individual MCFs using the MTLT-based sample preparation strategy proposed in the present study.

The in situ measurements presented here are inherently subject to beam-induced damage. In our organic samples with polymeric support films, the primary damage mechanism is radiolysis, while the effects of heating can be considered negligible. At an acceleration voltage of 300 keV, the dose rate for the in situ tensile testing was approximately 0.05 e^–^/A^2^, resulting in a total dose of 3 e^–^/A^2^. Moreover, we observed that upon activating the electron beam, the support film underwent homogeneous expansion, leading to prestretching of the MCFs and potentially gradual disruption of fibrillar adhesion to the support film. This homogeneous stretching persisted for approximately 10 s; thereafter, the stretching of the film ceased. However, fibril sliding against the support film resulted in relaxation compared to the initial prestrained state. This relaxation was confirmed by tracking the D-period of the MCF during static imaging (Figure S6). Notably, the D-period relaxation observed during static imaging was an order of magnitude lower than the relaxation detected during the application of quasi-static stretching to the MCF: 10 and 1–2 nm/s, respectively. While this effect represents a non-negligible influence on MCF behavior, the reported deformation behavior is presented without any adjustments to avoid introducing potential bias.

The in situ mechanical testing approach for individual MCFs within an electron microscope presents significant potential. Following our preliminary experiments, we are optimistic regarding the future directions this technique could pursue. As discussed above, one notable limitation of our testing methodology is that fibrillar stretching is influenced by adhesion to the supporting film, which complicates any precise estimates of local forces. For future investigations, it may be advantageous to explore alternative designs for the tensile holder. For instance, a copper tensile holder with multiple smaller milled windows as opposed to a single large central window could be implemented. Utilizing 2 × 2 μm windows would eliminate the necessity for an underlying support film, while the ends of the fibrils can be fixed directly to the tensile grid using focused ion beam deposition. Additionally, another potential strategy for conducting in situ tensile tests could involve a push-to-pull device, aiding the real-time recording of forces during the experiment. However, our attempts to employ this method indicated difficulties in drop-casting the fibrils onto the designated region without contaminating the device.

## Conclusions

This study employs mineralized turkey leg tendon as an excellent source for extracting mineralized collagen fibrils (MCFs) that closely resemble those found in bone. The drop-casting technique facilitates the successful transfer of individual MCFs onto TEM grids, allowing for detailed visualization of their native structural organization and composition through energy-dispersive X-ray spectroscopy and 4D-STEM imaging. Furthermore, our pioneering tensile tests provide compelling evidence that bone toughening mechanisms initiate at the nanoscale, with observable crack deflection occurring at the level of individual MCFs and remarkable tensile strains of at least 8%. These findings pave the way for future in situ mechanical testing using TEM, offering valuable insights into the mechanical properties of MCFs and potentially extending to other biological and architectured materials.

## Methods

### Sample Preparation

MTLT samples were prepared from turkey legs obtained from a local abattoir as part of the food chain. As these were post-mortem samples and no live animals were involved in the research, the study is exempt from formal ethical approval. Highly mineralized parts were dissected from the tendon bundles, mechanically cleaned from any soft tissue, and further cut with a surgical scalpel. The resulting tendon pieces of about 1.5 mm in diameter and 4.0 mm in length were rinsed with PBS solution, dab dried, and stored in the freezer at −20 °C. Before continuing sample manipulations, MTLT pieces were thawed in PBS solution (7.4 pH) for 24 h, split along the main tendon axis using a surgical scalpel, and ultrasonicated for 3 min in an Eppendorf with PBS. After that, the MTLT samples were transferred to Eppendorf tubes containing DI water to remove other noncollagenous components from the fibrils. The supernatant of the aqueous sample was examined under an optical microscope after drop-casting onto a heated Si wafer (∼30 °C). If the supernatant quality was insufficient, the sample was transferred to a new Eppendorf tube with DI water, and the process was repeated until the supernatant contained a sufficient concentration of collagen fibers and a minimal amount of impurities. The resulting solution with mineralized collagen fibrils (MCF) was dropcasted on (i) a copper TEM grid with lacey carbon films for imaging or (ii) a copper tensile stripe with nitrocellulose support film in the milled central window (Figure 2).

### TEM Techniques

Electron microscopes within the National Center for Electron Microscopy (NCEM) at Lawrence Berkeley National Laboratory were used in this study. STEM imaging and EDX mapping were carried out on an aberration-corrected FEI ThemIS microscope at 80 kV, which was equipped with a Gatan direct electron K2 camera and a Bruker SuperX EDS system, providing quantitative EDX maps with a nanometer resolution.

4D-STEM imaging was carried out at TEAM I, the double-aberration-corrected FEI Titan microscope at 300 kV with the Dectris Arina detector^72^ operating in full frame mode. The total dose per 4D-STEM scan varied between 0.43 e^–^/A^2^ and 3.5 e^–^/A^2^, depending on the scan resolution. Gold nanoparticles were used to measure the elliptical distortion and to calibrate the reciprocal-space pixel size.

### In Situ Tensile Tests

In situ tensile tests were performed on a TEAM I microscope with a Gatan single-tilt straining holder, model 654. The holder can elongate at a maximum rate of 2.0 μm s^–1^ with a nominal drift rate of 1.5 nm mm^–1^. The holder is purely displacement-controlled and does not measure the force.

Custom-made tensile stripes made of 0.5 mm thick copper foil with a milled hole in the center were used. The nitrocellulose support film was formed on the striped window by 2% collodion in an amyl acetate mixture. The MCF in solution was dropcast on the support film and imaged in situ during tensile tests.

### Image Processing and Quantification

EDX maps were analyzed in Velox v.3.17. The intensity profile for C, N, O, P, and Ca elements was corrected for the theoretically estimated *k*-factor and exported from each map to be analyzed in Python v.3.12. Intensity profiles were fitted with a linear combination of a sinusoidal and a linear function (scipy curve_fit)

1 f(x)=a+bx+csin(dx+e)

where *a* denotes the mean net intensity, and *d* is its periodicity, matching the physical D-period. The final D-period of each fibril is estimated as the mean *d* value from all elements. The Ca/P ratio is estimated as the ratio of the Ca/P mean intensities. The summary line plots with the fits for each fibril are shown in Figure S1.

4D-STEM analysis was carried out in Python v3.12 using the open-source py4DSTEM v0.14.17 software package.^73^ The analysis involved performing elliptical correction and calibration, followed by polar transformation. The angular position of the (002) diffraction spot was measured at each scan pixel in real space, with the resulting orientation vectors plotted as continuous lines on the real-space map, thereby illustrating the in-plane orientation of the crystallites. Their orientation is then compared to the in-plane orientation of the fibril extracted through the intensity gradient. A schematic summary of the 4D-STEM data processing is provided in Figure S3.

The D-period tracking of the MCF during in situ stretching was performed in Python, following the intensity profile fitting with eq 1, as described for the EDX data set. The initial zero-strain D-period was determined from a static imaging series (30 frames) of the region of interest acquired prior to deformation. This reference value was then used to calculate the strain within the MCF during crack propagation.

## Supplementary Material





## Data Availability

The data that support the findings of this study are available from the corresponding authors upon reasonable request.

## Notes

Summary of the EDX scans with the elemental intensity profile fitting and output correlation of the D-period vs Ca/P ratio; 4D-STEM data processing and summary; and in situ tensile tests (PDF)
In situ tensile test (MP4)
