# Supplementary material for: Structural and Mechanical Analysis of Individual Mineralized Collagen Fibrils Using In Situ Transmission Electron Microscopy
Source: ACS Nano. 2026 Mar 19;20(12):10127–37. doi: 10.1021/acsnano.6c00964 (PMC13045354; doi:10.1021/acsnano.6c00964)
Supplement: Supplementary file 1 [file nn6c00964_si_001.pdf]

Supporting Information

Structural and mechanical analysis of individual mineralized collagen fibrils using *in situ* transmission electron microscopy

Tatiana Kochetkova,[a] Stephanie M. Ribet,[b] Lilian M. Vogl,[bcd] Daniele Casari,[e] Rohan Dhall,[b] Philippe K. Zysset,[a] Andrew M. Minor,[bc] Peter Schweizer[bd*]*

[a] ARTORG Center for Biomedical Engineering Research, University of Bern, Bern CH 3010, Switzerland;

[b] National Center for Electron Microscopy (NCEM), The Molecular Foundry, Lawrence Berkeley National Laboratory, Berkeley, CA 94720, USA;

[c] Department of Materials Science and Engineering, University of California Berkeley, CA 94720, USA;

[d] Max Planck Institute for Sustainable Materials, Düsseldorf 40237, Germany

[e] Laboratory for Mechanics of Materials & Nanostructures, Empa - Swiss Federal Laboratories for Materials Science and Technology, Thun CH 3603, Switzerland;

* Corresponding Authors: tatiana.kochetkova@unibe.ch, p.schweizer@mpi-susmat.de

Supplementary sections:

1. Summary of the EDX scans with the elemental intensity profile fitting and the output correlation of the D-period vs Ca/P ratio (***Figure S1***, ***Figure S2***)
2. 4D-STEM data processing and summary (***Figure S3***, ***Figure S4***)
3. In situ tensile tests (***Figure S5, Figure S6***)

1. Summary of the EDX scans with the elemental intensity profile fitting and the output correlation of the D-period vs Ca/P ratio.

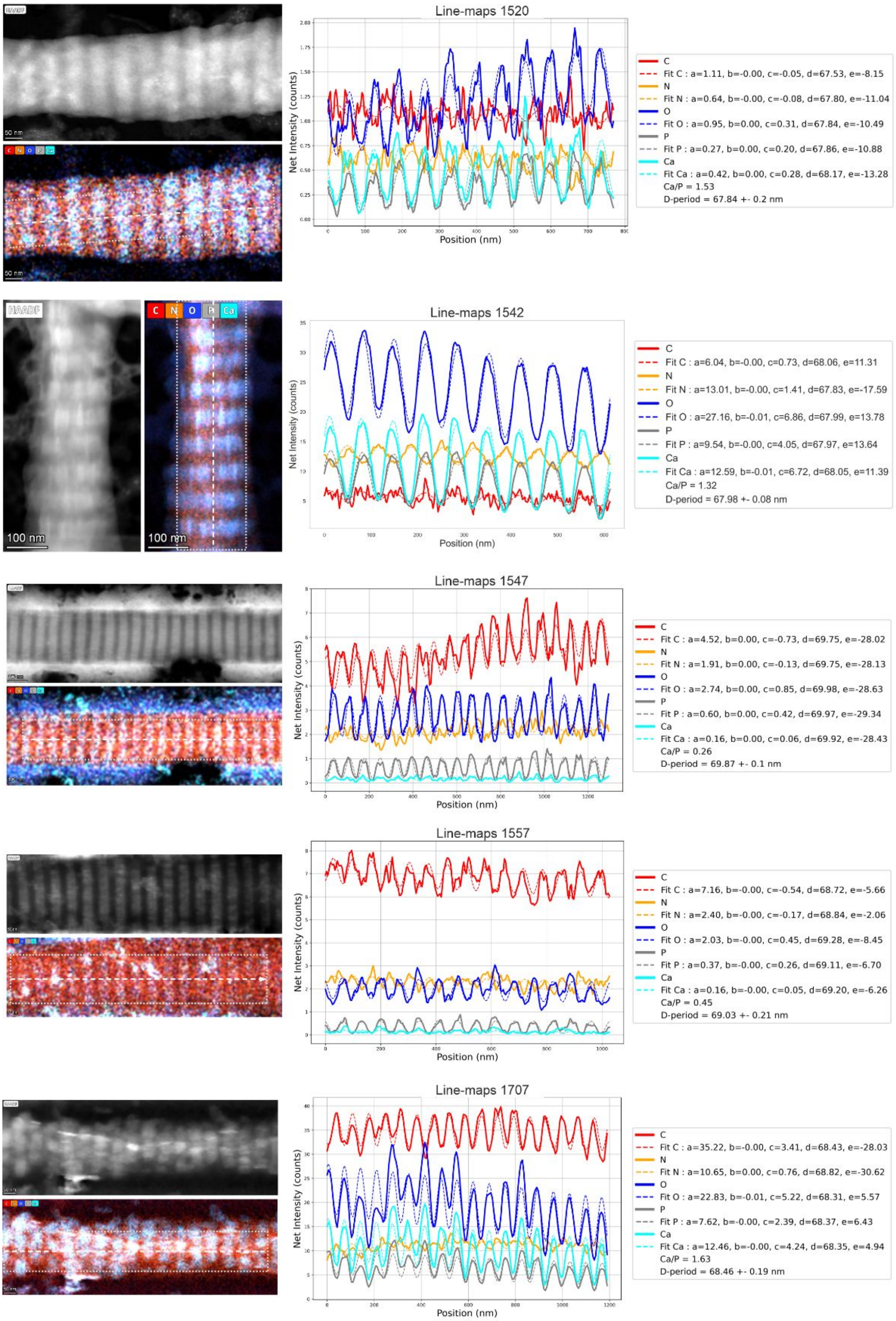

Figure S 1. HAADF images and corresponding EDX scans for five fibers with the elemental intensity profile fitting.

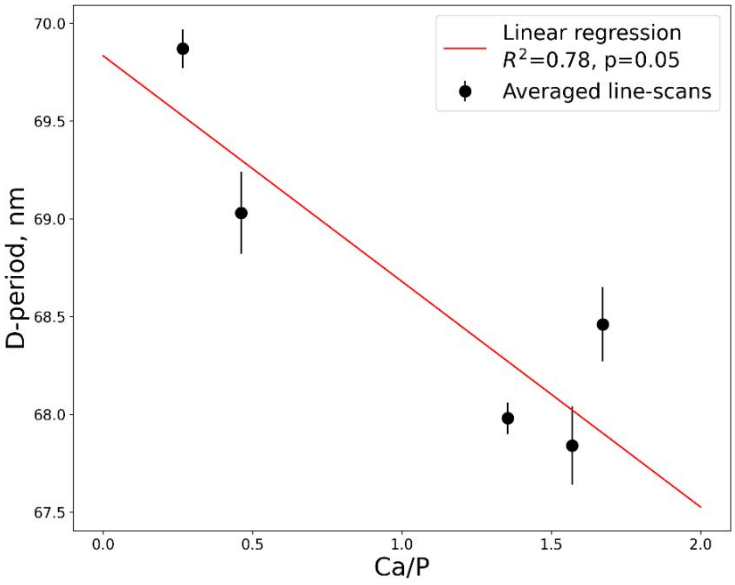

Figure S 2. D-period negatively correlates with the Ca/P ratio.

2. 4D-STEM data processing and summary

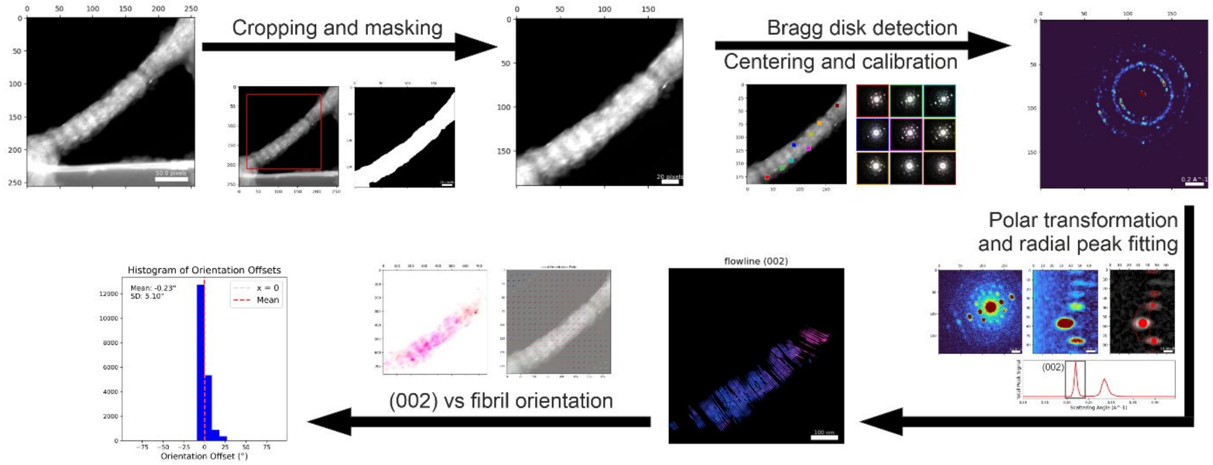

Figure S 3. 4D-STEM data processing pipeline.

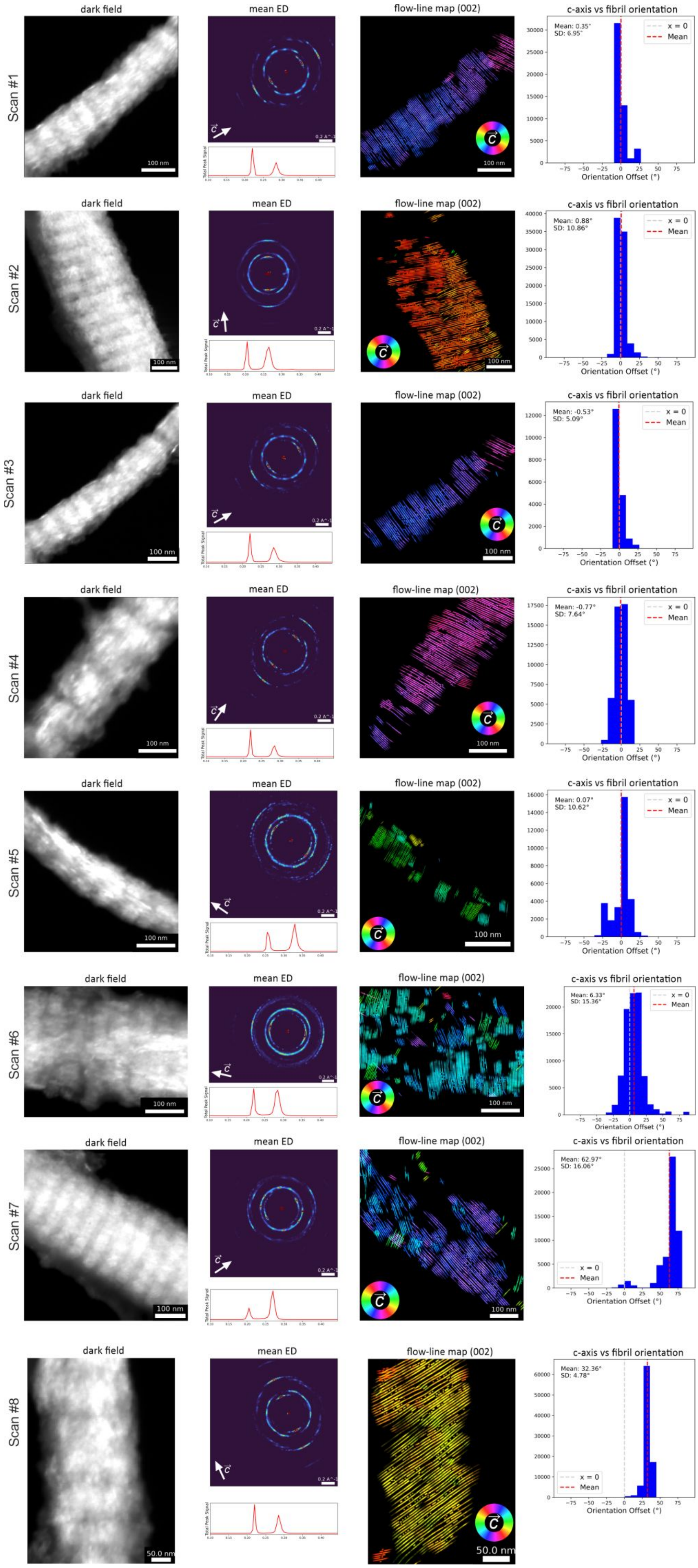
dark field
mean ED
flow-line map (002)
c-axis vs fibril orientation
Scan #1
100 nm
0.2 Å⁻¹
Mean: 0.35°
SD: 6.95°
x = 0
Mean
Orientation Offset (°)
Scan #2
Mean: 0.88°
SD: 10.86°
Scan #3
Mean: -0.53°
SD: 5.09°
Scan #4
Mean: -0.77°
SD: 7.64°
Scan #5
Mean: 0.07°
SD: 10.62°
Scan #6
Mean: 6.33°
SD: 15.36°
Scan #7
Mean: 62.97°
SD: 16.06°
Scan #8
50.0 nm
Mean: 32.36°
SD: 4.78°

Figure S 4. Summary of the flow line maps of all 4D-STEM scans, including (from left to right) reconstructed dark field image, mean electron diffraction and corresponding radial peaks, flow line map and resulting orientation variation between the fibril main axis and the c-axis of the mineral.

3. *In situ* tensile tests

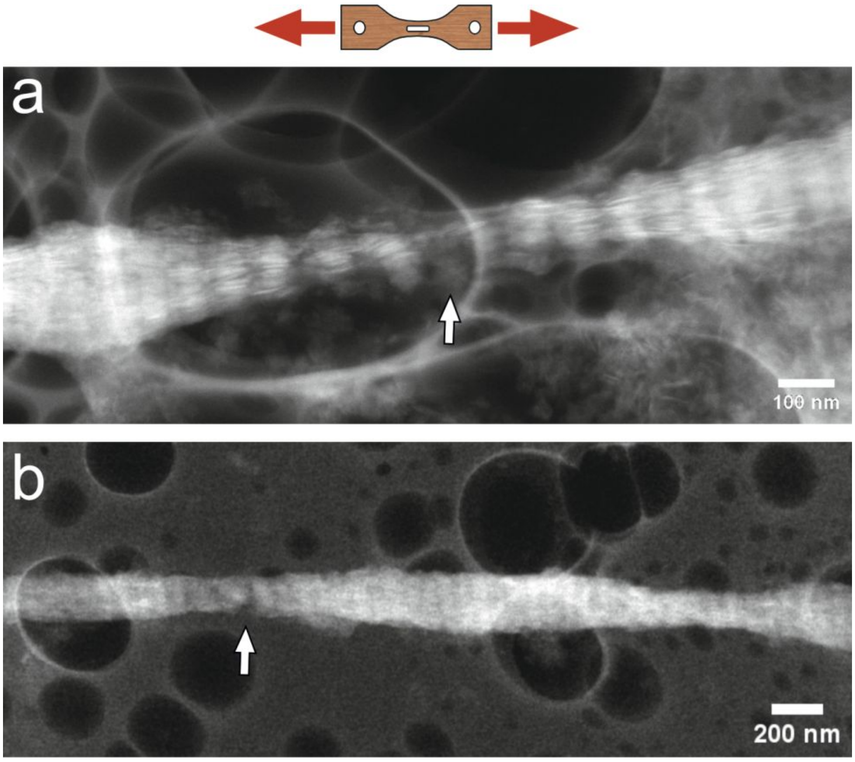

Figure S 5. HAADF images of two other MCFs that were subjected to tensile loads. Both fibrils demonstrate ductile deformation upon staining with visible D-period deterioration (a) and distorted crack propagation (b), marked with the white arrows. Note the deterioration of the support film due to electron beam exposure.

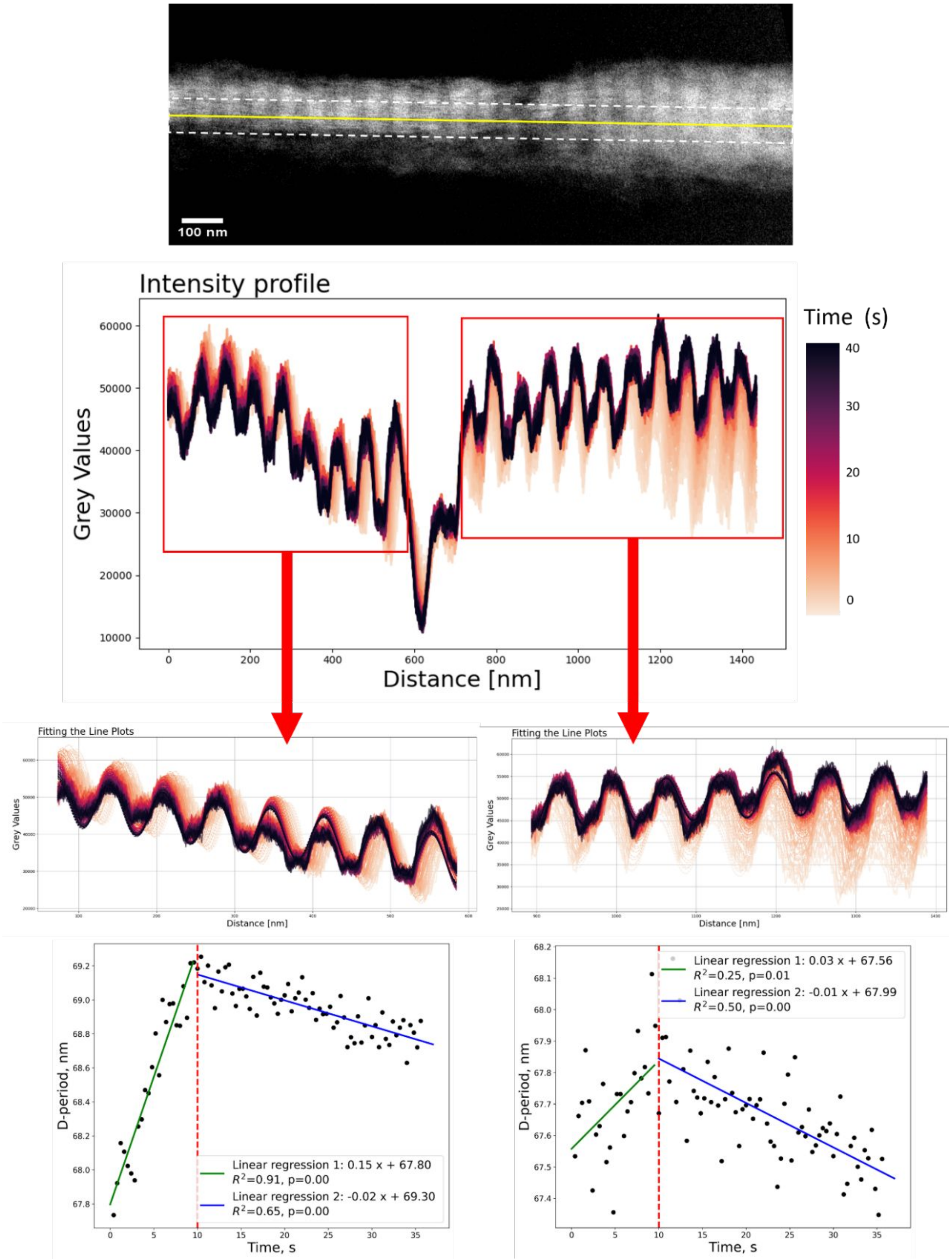

Figure S 6. In situ TEM imaging of the MCF without external displacement from the straining holder. Note the two phases of the MCF's D-period evolution: (1) pre-stretching due to the homogeneous expansion of the support film upon electron beam activation (linear regression 1), followed by (2) relaxation, likely caused by the cessation of film expansion and the MCF's consequent sliding against the support film (linear regression 2).
